# A Review of the Pathophysiology and Potential Biomarkers for Peripheral Artery Disease

**DOI:** 10.3390/ijms160511294

**Published:** 2015-05-18

**Authors:** Smriti Murali Krishna, Joseph V. Moxon, Jonathan Golledge

**Affiliations:** 1 The Vascular Biology Unit, Queensland Research Centre for Peripheral Vascular Disease, College of Medicine & Dentistry, James Cook University, Townsville, QLD 4811, Australia; E-Mails: smriti.krishna@jcu.edu.au (S.M.K.); joseph.moxon@jcu.edu.au (J.V.M.); 2 Department of Vascular and Endovascular Surgery, the Townsville Hospital, Townsville, QLD 4810, Australia

**Keywords:** peripheral artery disease, critical limb ischemia, biomarkers, angiogenesis, arteriogenesis

## Abstract

Peripheral artery disease (PAD) is due to the blockage of the arteries supplying blood to the lower limbs usually secondary to atherosclerosis. The most severe clinical manifestation of PAD is critical limb ischemia (CLI), which is associated with a risk of limb loss and mortality due to cardiovascular events. Currently CLI is mainly treated by surgical or endovascular revascularization, with few other treatments in routine clinical practice. There are a number of problems with current PAD management strategies, such as the difficulty in selecting the appropriate treatments for individual patients. Many patients undergo repeated attempts at revascularization surgery, but ultimately require an amputation. There is great interest in developing new methods to identify patients who are unlikely to benefit from revascularization and to improve management of patients unsuitable for surgery. Circulating biomarkers that predict the progression of PAD and the response to therapies could assist in the management of patients. This review provides an overview of the pathophysiology of PAD and examines the association between circulating biomarkers and PAD presence, severity and prognosis. While some currently identified circulating markers show promise, further larger studies focused on the clinical value of the biomarkers over existing risk predictors are needed.

## 1. Introduction

Narrowing or blockage of the arteries supplying blood to the lower limbs, usually termed peripheral artery disease (PAD), is principally caused by athero-thrombosis. PAD is a leading cause of morbidity due to the associated functional decline and limb loss. Both asymptomatic and symptomatic PAD are significant predictors of cardiovascular disease (CVD) events and mortality [1]. Current evidence suggests that PAD represents a CVD risk equivalent to or worse than coronary artery disease requiring aggressive medical management [2].

The main recognized clinical presentations of PAD are intermittent claudication (IC) and critical limb ischemia (CLI). IC describes the symptoms of pain in the muscles of the lower limb brought on by physical activity which is rapidly relieved by rest. CLI is a more severe manifestation of PAD, which presents as rest pain, ischemic ulceration or gangrene of the foot. Patients with CLI have a high risk of limb loss and fatal or non-fatal vascular events, such as myocardial infarction (MI) and stroke [2]. Acute limb ischemia (ALI) occurs when there is a sudden interruption of blood flow to a limb typically due to an embolism or thrombosis [3]. In contrast to CLI, which typically develops over a prolonged period often preceded by IC, patients with ALI may not have preceding symptoms. ALI usually threatens limb viability more urgently than CLI possibly due to the absence of an established collateral blood supply to the limb.

## 2. Epidemiology of PAD

The prevalence of PAD is estimated to be 10%–25% in people aged ≥55 years and increases to approximately 40% in community populations aged >80 years [4,5]. Approximately 4–8 million people are affected by PAD in the United States of America [6,7,8]. In Germany around 1.8 million people have symptomatic PAD and each year between 50,000 to 80,000 patients develop CLI [9,10]. In a population-based study in Western Australia, the prevalence of PAD was reported to be approximately 23% in men aged 75–79 years [4]. Recent reports suggest that the burden of PAD has increased globally over the last decade [11,12,13]. Atherosclerosis induced CLI has been associated with a mortality rate of 20%–25% in the first year after presentation and a survival rate of less than 30% at five years [14,15,16,17,18,19]. Previous reports suggest that CLI patients have a three-year limb loss rate of about 40% [20,21,22,23].

Recurrent CLI due to the failure of lower extremity revascularization is associated with a poor outcome [24,25]. In the Bypass *versus* Angioplasty in Severe Ischemia of the Leg (BASIL, *n* = 216) trial, the re-intervention rate in 216 patients with CLI treated by percutaneous transluminal angioplasty was 26% at 12 months [14]. Reasons for revascularization failure include restenosis, and residual and progressive atherosclerosis. Approximately 20%–30% of CLI patients are not ideal candidates for interventional procedures for a number of reasons such as the distribution of the occlusive disease and the patient’s co-morbidities [26]. Patients with CLI represent a small subset of the total PAD population however the high incidence of CVD events, repeated requirement for medical attention and high amputation rates lead to significant health service costs associated with these individuals [27,28]. Improvements in the current management of PAD are needed on many levels including earlier diagnosis, development of novel effective therapies and better application of currently available treatments.

## 3. Risk Factors for PAD

Approximately 70% of PAD cases can be explained by established risk factors such as older age, hypertension, dyslipidemia, cigarette smoking and diabetes [29]. It has been reported that for every 1% increase in hemoglobin A1c there is a corresponding 26% increase in PAD risk [30]. Insulin resistance has been identified as a risk factor for PAD even in subjects without diabetes [31]. The association between sex and PAD is less clear. The prevalence of asymptomatic or symptomatic PAD is slightly greater in men than in women and the incidence increases with increasing age [17]. Women have been reported to have a more advanced CLI stage at presentation (odds ratio (OR), 1.21; 95% confidence interval (CI): 1.21–1.23) [32]. Black ethnicity has been reported to increase the risk of PAD by over two fold [17]. There is also reported to be an association between underserved communities and major amputations due to PAD (adjusted OR, 1.29, 95% CI: 1.16–1.44) [33]. Cigarette smoking has been strongly associated with PAD incidence and heavy smokers have a four-fold higher risk of developing IC compared to non-smokers [17]. PAD thus shares risk factors with other CVDs, such as coronary artery disease, however some risk factors, such as smoking, are more powerfully associated with PAD than other CVDs.

## 4. Current PAD Management Strategies

PAD management focuses in part on the reduction of CVD risk factors [34,35]. A number of lower extremity performance measures have been suggested as prognostic markers in PAD and may be useful to identify patients at increased mortality risk [36]. Supervised exercise programs have been established as effective ways to increase pain free walking distance among patients with IC although they are not widely implemented [36]. Other medical therapies are applied to control pain, treat infection and promote ulcer healing. Revascularization, via endovascular means (e.g., percutaneous transluminal angioplasty or stenting) or open surgery (e.g., bypass), is the main treatment option for patients with CLI. Up to 30% of patients are not considered ideal for such interventions [26], the main reasons being unfavorable vascular involvement and peri-operative risk [37]. Previous studies suggest that endovascular and open surgical therapies provide similar outcomes for CLI. A recent meta-analysis of 23 studies reported no difference in amputation-free survival at three years (OR, 1.22, 95% CI: 0.84–1.77) and all-cause mortality (OR, 1.07, 95% CI: 0.73–1.56) in patients with CLI treated by endovascular or surgical revascularization [38].

There is current interest in the development of novel therapies to improve arteriogenesis (collateral formation) and/or angiogenesis (capillary formation) in patients with PAD [19]. A number of approaches are currently being investigated including gene therapies and cell-based therapies [26,39,40]. It was reported for example that intramuscular injection of autologous bone marrow mononuclear cells resulted in a three-year amputation free rate of 60% with significant improvement in ischemic leg pain and walking distance in the therapeutic angiogenesis by cell transplantation (TACT) trial [41]. In another recent trial, the use of tissue repair cells in patients with PAD (RESTORE-CLI), it was reported that administration of patient-derived bone marrow mononuclear cells led to an amputation free survival improvement of 32% [42]. These novel therapeutic approaches are promising strategies for CLI patients who are not ideal candidates for traditional revascularization procedures.

## 5. The Pathophysiological Response to Athero-Thombosis-Induced PAD

PAD is mainly caused by atherosclerosis and associated thrombosis within the lower limb arteries leading to end organ ischemia. Other causes include vasculitis and *in situ* thrombosis related to hypercoagulable states. The pathophysiology of athero-thrombosis induced PAD is complex, and involves a large number of cells, proteins and pathways. Important cells contributing to or controlling the development of athero-thrombosis include vascular endothelial cells (ECs), vascular smooth muscle cells (SMCs), fibroblasts, platelets, resident stem cells, pericytes and inflammatory cells [43,44,45]. The pathophysiology of atherosclerosis has been described in detail in previous reviews [46,47,48].

**Figure 1:**
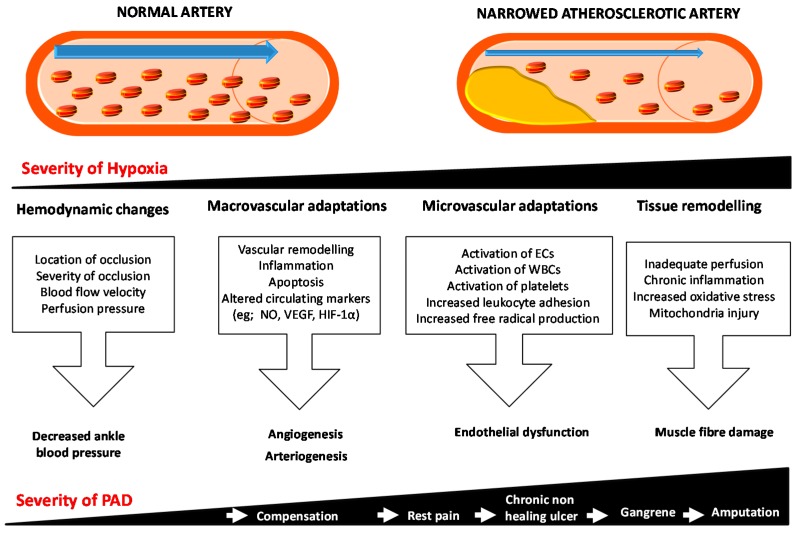
Schematic representation of the response to ischemia in peripheral artery disease. Initially the ischemic limb tries to compensate and resolve the hypoxia by changing the hemodynamics and promoting microvascular adaptations by promoting angiogenesis and/or arteriogenesis. As the severity of the hypoxia increases, the microvascular adaptations are not able to compensate. All these changes lead to mitochondrial injury and free radical generation and subsequent muscle fibre damage, myofibre degeneration and fibrosis. These changes eventually result in decreased oxygen supply and increased metabolic demands leading to conditions such as rest pain, chronic non-healing wounds and gangrene, subsequently threatening the limb function and viability. Blue arrows show the direction of blood flow in the artery and white arrows shows the increase in severity of disease. Abbreviations: ECs, endothelial cells; HIF-1α, Hypoxia inducible factor-1α; NO, Nitric oxide; PAD, Peripheral artery disease; VEGF, Vascular endothelial growth factor WBCs, white blood cells.

Under healthy conditions the response to gradually progressive limb ischemia involves the promotion of angiogenesis and arteriogenesis in an attempt to increase the blood supply to the affected limb. Vascular remodeling, inflammation and apoptotic pathways are also implicated in the ischemic response and these may in part contribute to the resolution of tissue damage. In patients with CLI these compensatory responses to ischemia are ineffective. As a result, there is on-going inadequate perfusion of tissue, endothelial dysfunction, chronic inflammation and high levels of oxidative stress. All these changes lead to mitochondrial injury, free radical generation, muscle fibre damage, myofibre degeneration and fibrosis, and tissue damage, which may present as gangrene [49,50,51,52,53] (Figure 1).

Arteriogenesis is the process of enlargement of pre-existing collateral arteries to contribute to tissue perfusion [54]. The primary driving force is the increased laminar blood flow or shear stress associated with redistribution of flow as a result of the reduction in downstream luminal pressure. The increase in shear stress promotes vessel enlargement [55], which is stimulated by activation of nitric oxide (NO) signaling and likely other signaling responses to flow [56,57]. Angiogenesis describes the development of new capillary networks. An important driving force for angiogenesis is tissue ischemia. In response to local hypoxia in the ischemic limb, sprouting of small endothelial tubes occurs from pre-existing capillary beds. A number of hypoxia-inducible growth factors play a role in this process, such as vascular endothelial growth factor (VEGF) and hypoxia inducible factor (HIF)-1α [58] (Figure 1).

Potential therapies designed to accelerate arteriogenesis and angiogenesis during ischemia are under intense investigation although most of these interventions have not developed to a stage that they are ready for widespread clinical use [59,60,61,62].

## 6. Potential Biomarkers for PAD

PAD is usually diagnosed using clinical assessment, ankle brachial pressure index (ABI) measurement or radiological imaging, or both, depending on the setting of the patient. Each of these approaches has advantages and disadvantages. ABI for example is a simple and cheap investigation to diagnose PAD but not applied frequently in routine practice [63,64]. Patients with IC alone typically have an ABI of 0.5–0.9, while CLI patients usually have an ABI of <0.4 [23]. Angiography provides detailed imaging of the lower limb arteries but it is more expensive and involves exposure to radiation. The introduction of circulating markers which can diagnose PAD or predict patient outcomes has been suggested as a means to overcome some of the limitations of current approaches.

A range of circulating markers of atherosclerosis, arterial stiffness, inflammation, angiogenesis, vascular extra cellular matrix (ECM) remodeling, endothelial dysfunction and oxidative stress have been reported to be associated with PAD. Some of these biomarkers have also been suggested to be useful in predicting disease severity and prognosis.

### 6.1. Circulating Markers Associated with the Presence of PAD

Case-control studies have reported the association of a number of circulating markers with PAD presence (Table 1).

**Table 1:** Examples of circulating biomarkers reported to be associated with peripheral artery disease.

Circulating Biomarkers Assessed	Sample Size (*N*)	Sample Studied	Association with PAD Presence	Refs.
B2M & cystatin C	CAD & PAD (197); CAD (81) & healthy controls (262)	Plasma	A biomarker panel comprising B2M, cystatin C, hsCRP and glucose were associated with PAD.	[65]
B2M, cystatin C, hsCRP & glucose	PAD (83) & controls (896)	Plasma	Levels of cystatin C and B2M but not hsCRP and glucose were significantly elevated in PAD patients.	[66]
aPWV, AIx & B2M	PAD (66) & healthy controls (66)	Plasma	B2M, aPWV and AIx were significantly increased in patients with PAD; among patients with PAD elevated B2M levels were independently associated with higher aortic stiffness.	[67]
hsCRP, fibrinogen & leukocyte count	The National Health and Nutrition Examination Survey 1999–2002 (4787 participants aged ≥ 40 years)	Blood	All 3 markers were independently associated with PAD.	[68]
hsCRP	PAD (82) & healthy controls (41)	Plasma	Increased hsCRP levels in PAD patients.	[69]
CD40 ligand, fibrinogen, Lp-PLA2 , osteoprotegerin, P-selectin, and TNF-R2, hsCRP, ICAM-1, IL-6, MCP-1 & MPO	Framingham Offspring Study participants (2800)	Plasma	IL-6 &TNF-R2 were associated with PAD independent of established risk factors.	[70]
VEGF-A, TNF-α & IL-8	PAD (130) & controls (36)	Serum	Lower VEGF-A and higher TNF-α & IL-8 in PAD patients.	[71]
High molecular weight & total adiponectin	PAD (110) & healthy controls (230)	Plasma	Lower adiponectin in women with PAD.	[72]
OxPL/ApoB & Lp-a	Men with PAD (143), women with PAD (144) & controls (432)	Plasma	Increased levels of OxPL/ApoB and Lp-a were associated with PAD.	[73]
Lp-PLA2 activity	PAD (172) & healthy controls (787)	Plasma	Increased Lp-PLA2 activity in PAD patients.	[74]
Lp-PLA2 level	PAD (145) & healthy controls (837)	Plasma	Lp-PLA2 levels were significantly associated with PAD.	[75]
NOx &, sNOX2-dp	PAD (50) & healthy controls (50)	Serum	NOX2 up-regulation is associated with artery dysfunction in PAD.	[76]
NO	PAD (82) & healthy controls (41)	Plasma	Increased NO levels & hsCRP levels in PAD patients.	[69]
TBARS & ICAM-1	PAD (31) & healthy controls (10)	Plasma	Increased in PAD.	[77]
Rho-kinase activity	PAD (40), combined CAD/PAD (40) & healthy controls (40)	Circulating leukocytes	Increased in PAD.	[78]
HCgp-39	PAD (316) & healthy controls (39)	Plasma	Median levels of HCgp-39 were significantly higher in PAD patients.	[79]
CD163 & TWEAK	PAD (155) & healthy controls (251)	Plasma	Decreased TWEAK level and higher sCD163 levels in PAD patients.	[80]
PON-3	PAD (118), CAD (72) & healthy controls (175)	Serum	Increased in PAD.	[81]
IL-6, E-selectin, MMP-2, MMP-9 & TGF-β1	PAD (80) & healthy controls (3076)	Plasma	Increased levels of IL-6, E-selectin, MMP-2 & MMP-9 and reduced levels of TGF-β1 in PAD patients.	[82]
sRAGE	PAD (201) & healthy controls (201)	Plasma	Decreased levels of sRAGE in PAD patients.	[83]
VEGF	PAD (293) & healthy controls (26)	Serum	Higher levels of VEGF in PAD patients.	[84]
Ang2, sTie2, VEGF, sVEGFR-1 & PlGF	PAD (46) & healthy controls (23)	Plasma	Levels of VEGF and sTie2 were significantly increased in PAD patients.	[85]
VEGF, PlGF & TSP-1	PAD (184) & healthy controls (330)	Plasma	Elevated TSP-1 levels associated with PAD.	[86]
VEGF-A, TNF-α & IL-8	PAD (130) & controls (36)	Serum	PAD patients have lower circulating VEGF-A and higher levels of TNF-α and IL-8.	[71]
EPCs, CD133, VEGFR-2, MDA-LDL & pentraxin-3	PAD (48) & healthy controls (22)	Serum & Plasma	EPCs and pentraxin-3 were increased in PAD patients; Cardiovascular events in PAD patients were associated with reduced EPC and increased MDA-LDL.	[87]
EPCs	PAD (45) & healthy controls (24)	Blood	The number and proliferative activity of circulating EPCs was significantly increased in PAD patients.	[88]

Abbreviations: AIx, Augmentation index; Ang2, Angiopoietin-2; Apwv, Aortic pulse wave velocity; B2M, β-2-microglobulin; CAD, Coronary artery disease; EPC, Endothelial progenitor cell; HCgp, Human cartilage glycoprotein; hsCRP, high-sensitivity C-reactive protein; ICAM, Intercellular adhesion molecule; IL, Interleukin; Lp-a, Lipoprotein-a; Lp-PLA2, Lipoprotein-associated phospholipase A2; MCP-1, Monocyte chemotactic protein 1; MDA-LDL, Malondialdehyde-modified low-density lipoprotein; MMP, Matrix metalloproteinase; MPO, Myeloperoxidase; NOx, Nitrite/nitrate; NO, Nitric oxide; OxPL/ApoB, Oxidized phospholipids on Apo-B100-containing lipoproteins; PAD, Peripheral artery disease; PlGF, Placenta growth factor; PON-3, Paraoxonase-3; sNOX2-dp, soluble nicotinamide adenine dinucleotide phosphate oxidase 2-derived peptide; sRAGE, Receptor for advanced glycation end products; sTie2, soluble Tyrosine kinase with immunoglobulin-like and EGF-like domains 2; sVEGFR, soluble VEGF receptor; TGF-β, Transforming growth factor-β; TNF, Tumour necrosis factor; TNFR2, Tumour necrosis factor receptor 2; TBARS, Thiobarbituric acid-reactive substances; TSP, Thrombospondin; TWEAK, Tumour necrosis factor-like weak inducer of apoptosis; VEGF, Vascular endothelial growth factor.

#### 6.1.1. Markers of Athero-Thrombosis and Inflammation

It was previously reported that a combination of β-2-microglobulin (B2M), cystatin C, high-sensitivity C-reactive protein (hsCRP) and glucose was associated with PAD [65,66]. Plasma B2M levels and parameters of arterial stiffness such as aortic pulse wave velocity and augmentation index have been reported to be significantly increased in PAD patients [67]. Some studies have shown that combining these circulating markers of inflammation and atherosclerosis with clinical parameters may improve the identification of PAD patients [65,66,71]. A previous nested case-control study reported an increased relative risk of PAD (2.8, 95% CI: 1.3–5.9) for subjects in the highest hsCRP quartile compared with the lowest quartile [89].

The levels of a number of lipids and fat related markers have also been reported to be differentially regulated in PAD patients. A recent lipidomic analysis comparing serum lipid profiles in 168 patients with PAD and 161 abdominal aortic aneurysm patients showed that a group of linoleic acid-containing triacylglycerols and diacylglycerols were negatively associated with PAD presence [90]. Furthermore, combining lipidomic features with traditional risk factors significantly improved stratification of patients with PAD and abdominal aortic aneurysm. The levels of total and high molecular weight adiponectin were reported to be significantly lower in women developing PAD [72]. Oxidized phospholipids (OxPL) on Apo-B100-containing lipoproteins (OxPL/ApoB) and the major lipoprotein carrier of OxPL, named lipoprotein-a (Lp-a), have been reported to be positively associated with risk of PAD [73]. Additionally, apolipoprotein (Apo)-A1 and high-density lipoprotein (HDL) have previously been associated with PAD and Apo-AI and homocysteine (Hcy) were reported to be predictors of ABI [91]. It should also be noted that up to 30% of PAD patients have elevated serum levels of Hcy in comparison to 1% of the general population [17]. Previous randomised trials have failed to identify any clinical benefit of folic acid and cobalamin supplementation even though this intervention resulted in a reduction in serum Hcy concentration [92]. There are however a number of ongoing clinical trials examining the benefits of such supplementation in PAD patients [92,93,94]. Patients with asymptomatic PAD have been reported to have a defined profile of pro-inflammatory markers with significantly higher levels of interleukin (IL)-6, E-selectin and matrix metalloproteinase (MMP)-2, MMP-9 and significantly reduced levels of transforming growth factor (TGF)-β1 [82].

Recently, a number of novel markers, such as Rho-kinase activity [78] and human cartilage glycoprotein-39 (HCgp-39/YKL-40) [79], have been highlighted as markers of atherosclerosis. The ratio between plasma levels of macrophage scavenger receptor CD163 and tumor necrosis factor-like weak inducer of apoptosis (TWEAK) was also shown to be a potential biomarker of athero-thrombosis in asymptomatic PAD subjects [80]. Since atherosclerosis is a systemic disease, most of these circulating markers may not be specific to PAD but rather reflect the underlying atherosclerotic burden. The paraoxonases (PON) retard lipoprotein oxidation and have been suggested to have anti-atherogenic properties including reducing low density lipoprotein oxidation, reducing oxidative stress, promoting reverse cholesterol transport from macrophages and normalizing vascular endothelial function. PON-1 activity has been suggested to modulate endothelial function in patients with PAD [95]. Circulating levels of PON-3 have been positively associated with B2M, chemokine ligand 2 (CCL2) and hsCRP in CAD, but not in PAD patients [81]. Since atherosclerosis is often widespread, it may be difficult to identify specific markers relating to PAD. Thus, markers that are altered in atherosclerosis in general might be of limited clinical application in identifying PAD patients specifically since most of them do not show a unique association with PAD.

#### 6.1.2. Markers of Oxidative Stress

An interesting observation was reported recently in relation to gender and racial differences in endothelial oxidative stress in patients with symptomatic PAD [96]. Female African American patients with symptomatic PAD were observed to have increased markers of oxidative stress and elevated levels of circulating pro-inflammatory biomarkers in comparison to males. The women also showed limited peripheral microcirculation, exercise performance, and ambulatory activity compared to male PAD patients. The findings were suggested to support the use of biomarkers related to oxidative stress in identifying symptomatic female PAD patients requiring interventions.

A number of reports suggest that markers of oxidative stress [97,98,99], such as reactive oxygen species (ROS), are consistently increased in patients with PAD. The bioavailability of ROS within the circulation depends on their rate of formation, by mitochondrial enzymes such as NADPH oxidase (NOX) and xanthine oxidase (XO), and their rate of clearance, through the antioxidant defense system [100]. Furthermore, the bioavailability of NO depends on the balance maintained between the formation of NO and its removal, which is partly dependent on the reaction of NO with ROS. NO is an important component of the complex system that regulates vascular resistance and blood flow distribution [101]. PAD is associated with decreased production of NO [102,103].

Nicotinamide adenine dinucleotide phosphate oxidase (NOX)-2, is a multicomponent enzyme and the catalytic core of NADPH oxidase. NOX-2 mediates electron transfer from NADPH oxidase to molecular oxygen, and is the most important producer of ROS. Compared to controls, patients with PAD have been reported to have enhanced soluble NOX-2-derived peptides, isoprostanes and reduced serum levels of NO and flow mediated dilation [76]. CLI patients have also been reported to have reduced levels of nicotinamide adenine dinucleotide (NADH) and increased levels of NAD+ within their gastrocnemius muscles [104]. These findings suggest that an altered ratio of NAD and NADH within the circulation might reflect a compromised metabolic and redox state in PAD patients. Furthermore, plasma levels of ROS components have been reported to be increased in IC patients [52,77].

#### 6.1.3. Markers of Vascular Remodeling

Reduced circulating concentrations of a number of proteins involved in ECM remodeling, such as TGF-β1, have been reported in PAD patients [82]. Another protein proposed to be a valuable biomarker of vascular inflammation, as well as PAD presence, is the soluble receptor for advanced glycation end products (sRAGE). It was reported that circulating concentrations of sRAGE were reduced in PAD patients [83]. Serum VEGF has been reported to be positively associated with PAD in one small study, however a larger study assessing plasma VEGF reported no association [71]. Plasma thrombospondin (TSP)-1 has also been reported to be upregulated in PAD patients [86]. Furthermore, TSP-1 has been reported to be expressed in newly formed vessels in PAD patients receiving local injections of bone marrow mononuclear cells and therefore could be reflective of therapeutic angiogenesis. *In vitro* and *in vivo* studies have suggested that recombinant human TSP-1 had a negative effect on angiogenesis. siRNA mediated TSP-1 inhibition has been reported to promote endothelial colony forming cells (ECFC) proliferation and recombinant human TSP-1 significantly enhanced ECFC adhesion [86]. Furthermore, a short peptide derived from the *N*-terminal part of TSP-1 named TSP-Hep-I also significantly enhanced the adhesion potential of ECFCs. Further work is needed to assess the value of circulating TSP-1 concentrations in identifying and providing prognostic information for PAD patients.

#### 6.1.4. Circulating Progenitor Cells

The number of circulating endothelial progenitor cells (EPC) has been reported to be associated with atherosclerosis and CVD however further work is needed to assess its value as a reliable biomarker for PAD [105]. It has, for example, been reported that compared to controls, the number of EPCs and the plasma pentraxin-3 concentration were increased in IC patients, but not in those with CLI [87]. In another study it was reported that cells expressing markers such as CD34^(+)^ or CD133^(+)^ circulated in lower numbers in PAD patients compared to healthy controls [88]. The cells expressing CD34^(+)^ or CD133^(+)^ are believed to represent bone marrow progenitors and studies in mice models have suggested they are involved in angiogenesis [80]. A thermal therapy using far infrared rays within a sauna (Waon therapy) has been reported to promote mobilization of CD34^(+)^ cells and augment ischemia induced angiogenesis in mice with hind limb ischemia [106].

Overall, while several biomarkers have been associated with PAD presence currently none of these markers have been consistently demonstrated to improve detection of PAD. In this context, markers of oxidative stress and circulating progenitor cell populations warrant further attention. Further investigations in which markers are examined in large and repeated groups of patients are needed to better examine the potential of using these circulating markers in clinical practice.

### 6.2. Markers Associated with the Severity and Outcome of PAD

Biomarkers may not only be useful in determining the presence or absence of a disease, but could also be useful in predicting patient outcomes. A detailed analysis of a reliable biomarker when combined with established risk factors could assist in stratifying patients at high risk and for selecting optimal treatment. Many published studies have assessed the value of prognostic biomarkers in small groups of patients. However, data that available markers can lead to consistent improvements in clinical management is currently not convincing. It is important that the associations between markers and prognosis observed in smaller isolated studies are followed up by validation in larger cohort studies in independent populations. This section of the review focuses on the most commonly reported circulating biomarkers linked to inflammation, endothelial dysfunction, angiogenesis and vascular remodeling and their relationship with PAD severity and outcomes such as reduced ABI, amputation and mortality (Table 2).

**Table 2:** Examples of circulating biomarkers reported to be associated with the severity and outcome of peripheral artery disease.

Circulating Biomarkers Assessed	Sample Size (*N*)	Sample Studied	Association with PAD Severity	Refs.
hsCRP, albumin, α-2 macroglobulin, fibrinogen, IL-1β, IL-1 receptor antagonist, IL-6, IL-6 receptor, IL-10, IL-18, TNF-α, & TGF-β	InCHIANTI study; PAD (955)	Serum	Higher levels of IL-1 receptor antagonist, IL-6, fibrinogen and hsCRP in PAD patients.	[107]
hsCRP, DD, TAT III & vWF	IC (132) & CLI (30)	Plasma	Higher levels of hsCRP, vWF, and TAT III in CLI compared to patients with IC.	[108]
hsCRP, fibrinogen & SAA	PAD (91)	Plasma	hsCRP, fibrinogen, and SAA levels were significantly associated with CLI; elevated hsCRP correlated with adverse graft-related or cardiovascular events.	[109]
hsCRP, DD, IL-6, VCAM-1, ICAM-1 & Hcy	Walking and Leg Circulation Study (WALCS); PAD (423)	Serum	Higher levels of inflammation markers and DD were associated with poorer lower extremity performance.	[110]
hsCRP, DD, SAA & fibrinogen	PAD (337)	Serum	Elevated baseline levels of inflammatory markers and DD were associated with greater decline in the physical performance.	[111]
hsCRP	PAD (225)	Plasma	A risk prediction model including hsCRP combined with traditional risk factors, renal function, and nutrition had excellent discriminatory ability in predicting all-cause mortality in patients with clinically advanced PAD undergoing bypass surgery.	[112]
hsCRP	PAD (118)	Plasma	Increased pre-procedural hsCRP levels were associated with major adverse limb events and late cardiovascular events.	[113]
hsCRP	Hemodialysis patients undergoing endovascular therapy for PAD (234)	Serum	Elevated pre-procedural hsCRP levels were associated with re-intervention or above ankle amputation and any-cause death after endovascular therapy.	[114]
hsCRP	European Prospective Investigation into Cancer and Nutrition (EPIC)-Norfolk cohort; Healthy participants (18,450)	Serum	In the EPIC-Norfolk cohort, hsCRP was associated with nonfatal PAD events.	[115]
hsCRP, LDL & HDL	Total PAD (100); IC (73)	Blood	Walking disability in PAD was associated with arterial endothelial dysfunction; Endothelial dysfunction was more significantly associated with walking disability in IC.	[116]
ApoA-I, HDL, Hcy, folate & vitamin B12	Elderly volunteers from rural Sicily (667)	Serum	Decreased ApoA-I and increased Hcy were predictors of ABI.	[91]
VCAM-1, ICAM-1 & MCP-1	PAD (112)	Serum	Increased sVCAM-1 and sICAM-1 were associated with PAD.	[117]
ICAM-1, leptin, Apolipoprotein-CIII	PAD (148)	Serum	African American women with symptomatic PAD had an increased oxidative stress related markers compared with men.	[96]
MPO	PAD (406)	Plasma	Plasma level was useful for risk stratification of PAD.	[118]
TRAP-6-inducible P-selectin expression	PAD (108)	Blood	Low thrombin generation potential was associated with an 11.7-fold increased risk of future atherothrombotic events.	[119]
Galectin-3	CLI (55)	Serum	Increased levels of Galectin-3 in CLI.	[120]
NT-pro-BNP	PCA (100), PAD (300) & healthy controls (300)	Serum	Patients with PCA had higher levels of NT pro-BNP than PAD and controls suggestive of an adverse hemodynamic milieu and increased risk for adverse cardiovascular outcomes.	[121]
NT-pro-BNP	PAD (481)	Serum	Higher levels of NT-pro-BNP were independently associated with a lower ordinal walking category or functional capacity.	[122]
Ang2, Tie2, VEGF, VEGFR-1 & PlGF	PAD (46) & healthy controls (23)	Plasma	Levels of VEGF and sTie2 were significantly increased in CLI.	[85]
VEGF-A 165b	PAD (18)	Serum	Increased anti-angiogenic VEGF-165b and a corresponding reduction in levels of the pro-angiogenic VEGF-A165a.	[123]

Abbreviations: ALI, Acute limb ischemia; Ang2, Angiopoietin-2; CLI, Critical limb ischemia; DD, D-Dimer; Hcy, Homocysteine; HDL, High-density lipoprotein; hsCRP, high-sensitivity C-reactive protein; IC, Intermittent claudication; ICAM, Intercellular adhesion molecule; IL, Interleukin; LDL, Low-density lipoprotein; MCP-1, Monocyte chemotactic protein 1; MPO, Myeloperoxidase; MMP, Matrix metalloproteinase; NO, Nitric oxide; NT-pro-BNP, *N*-terminal pro-B-type natriuretic peptide; PAD, Peripheral artery disease; PCA, Poorly compressible arteries; PlGF, Placenta growth factor; SAA, serum amyloid A; TAT III, Thrombin-antithrombin III; TGF-β, Transforming growth factor-β; TIMP, Tissue inhibitor of matrix metalloprotenase; TNF, Tumour necrosis factor; VCAM, Vascular adhesion molecule; VEGF, Vascular endothelial growth factor; vWF, von Willebrand factor.

#### 6.2.1. Markers of Inflammation

Multiple studies have reported that inflammation associated biomarkers circulate at higher levels in PAD patients with more severe disease [68,70,107,108,109,124] (Table 2). Cholesterol undergoes oxidation via both enzymatic stress-driven and free radical-mediated mechanisms and generates a range of oxysterols. Accumulation of hydroxycholesterols (HC) such as 25-HC, 27-HC and 24S-HC in the aortic intima was associated with systemic inflammatory activity and advanced atherosclerotic disease in individuals with severe PAD [125]. In the National Health and Nutrition Examination Survey of American men and women aged ≥40 years (*n* = 4787), elevated levels of hsCRP, fibrinogen, and leukocyte counts were associated with lower ABI values [68]. High levels of the inflammatory markers IL-6, hsCRP, soluble vascular adhesion molecule-1 (sVCAM-1) and soluble intercellular adhesion molecule-1 (sICAM-1) have been reported to be associated with accelerated functional decline in PAD patients [69,82,110,111,117] and with PAD complications such as amputation or general complications such as MI, stroke and death (Table 2). A risk prediction model was developed in a prospective cohort study of patients with clinically advanced PAD undergoing lower extremity bypass surgery (*n* = 225, followed up for a median of 893 days). The study showed that the traditional risk factors and circulating markers of inflammation such as hsCRP, sVCAM-1, renal function, and nutrition had excellent discriminatory ability in predicting all-cause mortality in PAD patients [112]. hsCRP has been shown to be an independent predictor of adverse cardiovascular outcomes and increased risk of secondary interventions or limb loss in PAD patients [126]. Elevated pre-procedural serum levels of hsCRP have been associated with requirement for re-intervention or above ankle amputation and mortality after endovascular therapy in haemodialysis patients with PAD [114]. Similar findings have been reported in other PAD patient groups [113]. A recent large prospective cohort study involving 18,450 participants (European Prospective Investigation into Cancer and Nutrition (EPIC)—Norfolk cohort) reported a strong and independent association between elevated hsCRP levels and an increased risk of PAD complications [115].

#### 6.2.2. Markers of Oxidative Stress and Endothelial Damage

Myeloperoxidase (MPO) is a peroxidase enzyme stored in neutrophils and a key mediator of inflammatory and redox-dependent processes in atherosclerosis. MPO has been reported to promote atherosclerosis via inducing oxidative stress in animal models [127,128]. A previous study suggested the potential value of using plasma MPO for risk stratification of major adverse events in PAD patients [118]. Similarly, *in vitro* thrombin generation potential was reported to correlate inversely with protease-activated receptors (PAR)-1-mediated platelet activation and was linked to the occurrence of athero-thrombotic events in patients with PAD [119]. The protein Galectin-3 is a soluble β-galactoside-binding lectin protein that has been implicated in athero-thrombosis and suggested as a biomarker for heart failure [129]. A five-year follow-up study of 309 PAD patients reported that circulating Galectin-3 concentrations were significantly and independently associated with an increased risk for CVD mortality (OR = 2.24, 95% CI: 1.06–4.73; *p* < 0.05) suggesting its potential use as a prognostic marker [130].

Arterial occlusion results in inadequate oxygen supply to the lower extremities and concurrent defective clearance of toxic metabolites. The net result is the accumulation of ROS, leading to tissue necrosis and microcirculatory damage that eventually result in irreversible deterioration and injury to the endothelial lining [131,132]. A previous study reported that reduced walking ability in PAD patients was associated with arterial endothelial dysfunction [116]. In another study however circulating concentrations of the endothelial marker NO were not associated with the severity of PAD [69]. Plasma levels of ROS markers, such as thiobarbituric acid-reactive substances (TBARS), sICAM-1 and antioxidants were increased in IC patients indicating that increased ROS production and damaged electron transport chain complexes may contribute to PAD and may serve as potential markers for its severity [52,77,133].

A recent report suggests that serum *N*-terminal pro-B-type natriuretic peptide (NT-pro-BNP) levels are associated with functional capacity in patients with PAD and may be a marker of hemodynamic stress in these patients [122]. Natriuretic peptides are mainly secreted from the heart in response to increased wall stress and NT-pro-BNP is elevated in patients with increased left ventricular mass and coronary heart disease, in addition to PAD [134]. Higher levels of circulating NT-pro-BNP have been associated with increased CVD mortality in PAD patients [113], and also patients with CHD [135,136]. Serum NT-pro-BNP levels were reported to be significantly higher in patients with poorly compressible arteries (PCA) than in those with PAD [121]. PCA occur due to medial arterial calcification and patients with PCA have an elevated ABI. Medial arterial calcification is associated with a number of chronic disease conditions and an increased risk predictor for CVD events and lower extremity amputation [137]. Even though medial arterial calcification and intimal calcification (which is a hallmark of atherosclerosis) may coexist, these two conditions are suggested to be distinct [138]. A recent report on the histopathological assessment of PAD (176 upper and lower leg artery specimens) patients highlights that the most common observations were medial calcification (present in 72% of arteries examined) and intimal thickening without lipid (present in 68% of arteries examined). Classical atherosclerosis was only identified in 23% of arteries [139]. Non-atheromatous intimal thickening was frequently observed, resulting in complete occlusion in some vessels. This suggests that vascular lesions in PAD patients may have additional pathological mechanisms other than atherosclerosis alone.

#### 6.2.3. Markers of Vascular Remodeling

One of the most studied markers of angiogenesis in PAD is VEGF and its receptors R1 and R2. VEGF-R1 is characteristically up-regulated by hypoxia and its soluble form has been proposed as a marker for CLI. Patients with advanced PAD have been reported to have elevated serum and tissue levels of VEGF compared to age matched healthy volunteers [84,85]. Furthermore, VEGF values in Fontaine class IV patients have been reported to be almost double that in healthy controls. High circulating VEGF levels have been associated with poor outcomes, such as major amputation, suggesting serum VEGF to be a potential marker for PAD severity. These findings could be interpreted as suggesting that administration of VEGF in an attempt to achieve therapeutic angiogenesis for CLI may not be safe [140]. It is however likely that the association of high VEGF concentrations with poor outcome reflects reverse causality as a response to the severity of ischemia rather than a cause.

Another, member of the VEGF family is VEGF-A, which exists as several isoforms produced as a result of alternative splicing. Reduced levels of circulating levels of total VEGF-A have been observed in IC patients compared to healthy controls [71]. Lower levels of total VEGF-A have also been associated with a reduction in capillary to muscle fibre ratio in PAD subjects [141]. A recent report suggested that in PAD, vascular insufficiency occurs in spite of elevated levels of total VEGF-A [123]. However, further analysis showed that PAD was associated with elevated levels of a VEGF-A splice isoform, VEGF-A165b and a corresponding reduction in levels of the VEGF-A165a splice isoform. Further studies in a rodent model suggested that VEGFA-165b had anti-angiogenic properties and the VEGF-A165a isoform was pro-angiogenic. Studies in a mouse model of PAD suggested that administration of VEGF-A165b inhibited revascularization [123]. Additional studies are warranted in patients in different PAD stages to understand the use of various VEGF-A isoforms as markers of angiogenesis status. Higher levels of plasma angiopoietin-2 (Ang2), and soluble Tie2 (sTie2) have been reported in patients with CLI compared to those with IC [85], indicating that there are other potential circulating biomarkers of disease severity that warrant attention.

The ECM plays a major role in regulating angiogenesis and *in vitro* and *in vivo* studies suggest a crucial role of proteolytic enzymes and matrix proteins in creating a permissive microenvironment to promote blood vessel sprouting [142,143,144]. Reorganization of the ECM, promoted by MMPs, creates space required for expansive remodeling of the pre-existing collaterals. Circulating levels of MMP-2, MMP-9, MMP-19 and Tissue inhibitor of metalloproteinase (TIMP)-1 have been reported to be elevated in CLI patients [74,82,145]. Furthermore, elevated plasma levels of MMP-2 and MMP-9 have been associated with PAD severity [146]. A previous study reported that the levels of both MMP-2 and MMP-9 (both the latent and the active forms) significantly increased during the active phase of limb reperfusion [147]. This temporal increase in MMP activity coincided with enhanced exposure of the unique cryptic collagen epitope (HU177) which has been previously implicated in angiogenesis in ischemic muscle. These findings suggest an important role for collagen remodeling during the active phase of ischemic limb reperfusion. Furthermore, studies in an animal model suggest that MMP-9 is essential for ischemia induced neovascularization by modulating bone marrow derived EPCs. MMP-9 deficiency impairs ischemia-induced neovascularization, through a reduction in EPC mobilization, migration, and vasculogenesis functions [148]. Additionally, use of ischemic muscle markers is only likely to be practical clinically if circulating forms of these markers are reflective of those in the muscle. It is not currently clear whether any reliable relationship exists between blood and muscle marker levels.

## 7. Conclusions

In this review, we have presented an overview of markers associated with PAD presence and outcome. A number of limitations exist for circulating markers currently identified. There is little consensus on which markers are consistently associated with PAD and there are definite challenges in identifying markers that are PAD-specific. The circulating markers should ideally reflect the changes occurring in the ischemic lower-limb muscles. In order to have clinical utility, the circulating biomarkers should predict risk independently of other established PAD risk factors. Furthermore, the circulating marker should be specific and sensitive and easily assayed.

**Figure 2:**
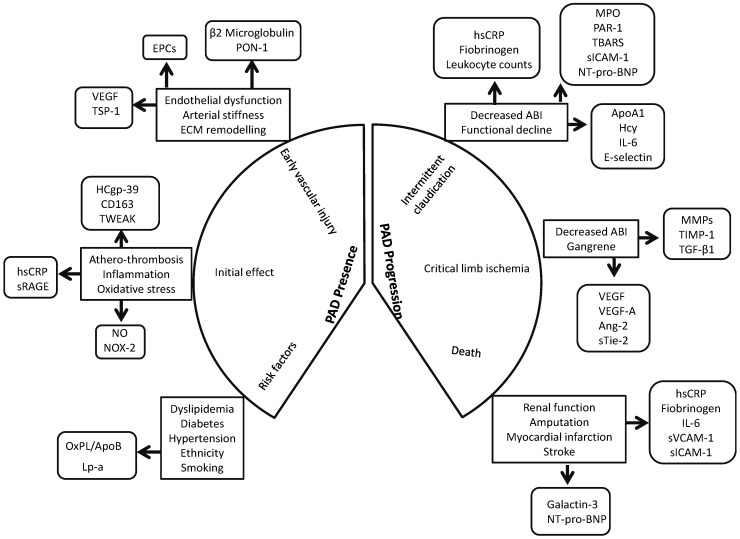
Circulating biomarkers in peripheral artery disease. A schematic depiction of the stages at which circulating biomarkers could be informative in the peripheral artery disease (PAD) course. Since PAD is multifactorial it is likely that a single biomarker may not be sufficient to predict diagnosis or prognosis. Since PAD development and progression is due to the interaction of multiple factors, it is possible that the combination of a number of biomarkers may be preferable to a single maker. Abbreviations: ABI, Ankle brachial index; Ang-2, Angiopoetin-2; ApoA1, Apolipoprotein A1; B2M, β-2-microglobulin; EPC, Endothelial progenitor cell; HCgp, Human cartilage glycoprotein; Hcy, Homocysteine; hsCRP, high sensitivity C-reactive protein; IL, Interleukin; Lp-a, Lipoprotein-1; MMP, Matrix mettalloprotenase; MPO, Myeloperoxidase; NO, Nitric oxide; NOX, NADPH Oxidase; NT-pro-BNP, *N*-terminal pro-B-type natriuretic peptide; OxPL/ApoB, Oxidised phospholipids on ApoB100 containing lipoproteins; PAD, Peripheral artery disease; PAR, Protease activated receptor; PON, Paraoxonase; sICAM-1, soluble Intercellular adhesion molecule-1; sRAGE, soluble receptor for advanced glycation end product; sTie-2, soluble Tyrosine kinase with immunoglobulin-like and EGF-like domains 2; sVCAM-1, soluble Vascular cell adhesion molecule-1; TBARS, Thiobarbituric acid-reactive substrates; TGF, Transforming growth factor; TWEAK, Tumour necrosis factor like weak inducer of apoptosis; TSP, Thrombospondin; TIMP, Tissue inhibitor of matrix metalloproteinase; VEGF, Vascular endothelial growth factor.

Biomarkers that could effectively predict PAD development, progression and outcome could be extremely useful in the clinical setting [149,150,151]. Ideally such markers would also identify patients best suited to different types of treatment. Currently, however, there is no convincing evidence that the markers investigated are reliable and specific. Even though novel markers such as Rho-kinase activity, human cartilage glycoprotein-39, TWEAK and PON-3, have been associated with PAD presence, their validity as reliable marker is unclear as these individual reports are frequently not followed by other replication studies. Some markers of inflammation, such as B2M, hsCRP and interleukins, oxidative stress, such as NO and NOX-2, ECM remodeling, such as TGF-β1, TSP-1 and VEGF, and circulating progenitor cell populations have been consistently associated with PAD presence (Figure 2). Similarly, markers of inflammation, such as hsCRP and sVCAM-1, oxidative stress, such as MPO and NT-pro-BNP, ECM remodeling, such as VEGF, VEGF-A isoforms and MMPs, have been consistently associated with PAD severity. Since cell therapy is a promising approach in PAD management, assessment of circulating stem cell populations may be of potential value. A comprehensive characterization of the circulating progenitor cell markers may be useful in identifying patients suitable for cell therapies. It should also be noted that initial investigations often over-estimates the effect size of a discovered association and thus it is important to re-assess findings in different populations. Thus it is important that prospective studies using large patient populations are conducted to validate biomarkers of interest to gain insight into their “real-world” benefit as diagnostic or prognostic aids.

In summary: (1) PAD is multifactorial; therefore single biomarker may not be powerful enough for reliable diagnosis and/or prognosis. Rather a combination of risk factor and circulating marker data may be needed; (2) Identifying a marker specific to PAD rather than general athero-thrombosis may be technically challenging; (3) Despite considerable effort, no biomarkers have been currently shown as sufficiently robust to be incorporated into clinical practice. Even though a number of currently identified circulating markers show promise, further larger studies focused on the clinical value of such markers over existing risk predictors are needed.
